# Consideration of the Management of Pediatric Fever Clinics During the Novel Coronavirus Pneumonia Outbreak

**DOI:** 10.1017/dmp.2020.289

**Published:** 2020-08-12

**Authors:** Hui Ding, Zhaoling Shi, Zhen Ruan, Xiaoning Cheng, Ruying Li, Lu Zhang, Xuanming Han, Zhihong Zhang, Nairong Gao, Yanzi Guo, Meng Zhang, Wenjuan Ma, Ziquan Liu, Guocheng Zhang

**Affiliations:** Children’s Hospital, The Second Affiliated Hospital of Shaanxi University of Chinese Medicine, Xianyang, China; Shaanxi University of Chinese Medicine, Xianyang, China; School of Disaster Medical Research, Tianjin University, Tianjin, China

**Keywords:** children, management, novel coronavirus pneumonia, pediatric fever clinics

## Abstract

Since the outbreak of 2019 novel coronavirus (2019-nCoV) infection in Wuhan City, China, pediatric cases have gradually increased. It is very important to prevent cross-infection in pediatric fever clinics, to identify children with fever in pediatric fever clinics, and to strengthen the management of pediatric fever clinics. According to prevention and control programs, we propose the guidance on the management of pediatric fever clinics during the nCoV pneumonia epidemic period, which outlines in detail how to optimize processes, prevent cross-infection, provide health protection, and prevent disinfection of medical staff. The present consideration statement summarizes current strategies on the pre-diagnosis, triage, diagnosis, treatment, and prevention of 2019-nCoV infection, which provides practical suggestions on strengthening the management of pediatric fever clinics during the nCoV pneumonia epidemic period.

## INTRODUCTION

Novel coronavirus pneumonia (NCP) was named by the World Health Organization as *coronavirus disease* (COVID-19),^1-4^ and its pathogen is 2019 novel coronavirus (2019-nCoV) belonging to the β-coronavirus genus. The International Committee on Taxonomy of Viruses (ICTV) renamed 2019-nCoV as *severe acute respiratory syndrome coronavirus 2* (SARS-CoV-2), whose genetic features are significantly different from severe acute respiratory syndrome-related coronavirus (SARSr-CoV) and the Middle East respiratory syndrome-related coronavirus (MERSr-CoV), but more than 85% homologous to bat SARS-like coronavirus (bat-SL-CoVZC45).^1,2,4-8^ Patients infected by 2019-nCoV are the main source of infection. Asymptomatic individuals are also an important source of infection and are easily overlooked in controlling transmission.^2,5,6,8^ Respiratory droplets and direct contact are the conventional routes of transmission, and it can also be spread through conjunctiva of the eyes.^9^ Aerosol and fecal-oral transmission are yet to be confirmed.^2,5,6,8^ The incubation period of 2019-nCoV infections usually ranges from 3 to 7 days, and the longest can reach 24 days.^8^ The infectivity of SARS-CoV-2 is significantly stronger than SARSr-CoV. Therefore, it’s extremely difficult to control the prevalence of 2019-nCoV.

People of all ages are susceptible to 2019-nCoV.^10-12^ Recently, pediatric cases have gradually increased. Most of them are infected by parents or caregivers. Children with NCP are common in family cluster outbreak, and critical cases have been reported.^8^ The youngest infected is a newborn after the birth of 36 hours.^2,5,6^ Research has shown that children are at similar risk of infection as the general population. Among children under the age of 10 who may have been exposed to SARS-CoV-2, approximately 7% and 8% of contacts in known cases subsequently tested positive. During the outbreak, how to implement national scientific prevention and control, carry out precise strategies, realize early detection, early diagnosis, early reporting, early isolation, early treatment, and take more practical and effective methods to prevent 2019-nCoV cross-infection and completely block dissemination are major subjects and a responsibility for medical workers in the febrile children diagnosis process.^4^ Therefore, a consideration statement summarizes current strategies on pre-diagnosis, triage, diagnosis, treatment, and prevention of 2019-nCoV infection, which provide practical suggestions on strengthening the management of pediatric fever clinics during the NCP epidemic period.

## IMPORTANCE OF STRENGTHENING THE MANAGEMENT OF FEBRILE CHILDREN DIAGNOSIS PROCESS

During the outbreaks, due to the strong infectivity of 2019-nCoV, pediatric fever clinics are prone to becoming the source of cross-infection and virus spread. In particular, winter and spring are the seasons of high incidence of respiratory diseases in children, and the symptoms of common respiratory diseases are similar to 2019-nCoV infection, which significantly increases the risk of cross-infection in children and infection in medical staff during the visit. At the same time, with the weather getting better and the normal operation of outpatient service, the flow of patients will increase remarkably, then suspected patients, common fever patients, and accompanying parents are mixed, which is prone to cause cross-infection and virus spread. At present, the pediatric departments of different hospitals have adopted various procedures for febrile children diagnosis according to their own conditions. However, some have obvious defects and hidden trouble. Therefore, before the end of epidemic, it is particularly necessary to strengthen the management of the febrile children diagnosis process, which is of great significance for the early detection of suspected children, prevention of cross-infection, stopping transmission, and, finally, eliminating the epidemic completely.

## MEASURES TO STRENGTHEN MANAGEMENT OF FEBRILE CHILDREN OUTBREAKS AND PREVENT CROSS-INFECTION

### Reinforce the Pre-Examination and Triage Process, and Identify Suspected Cases Early

#### Primary Pre-Examination and Triage

**FIGURE 1:**
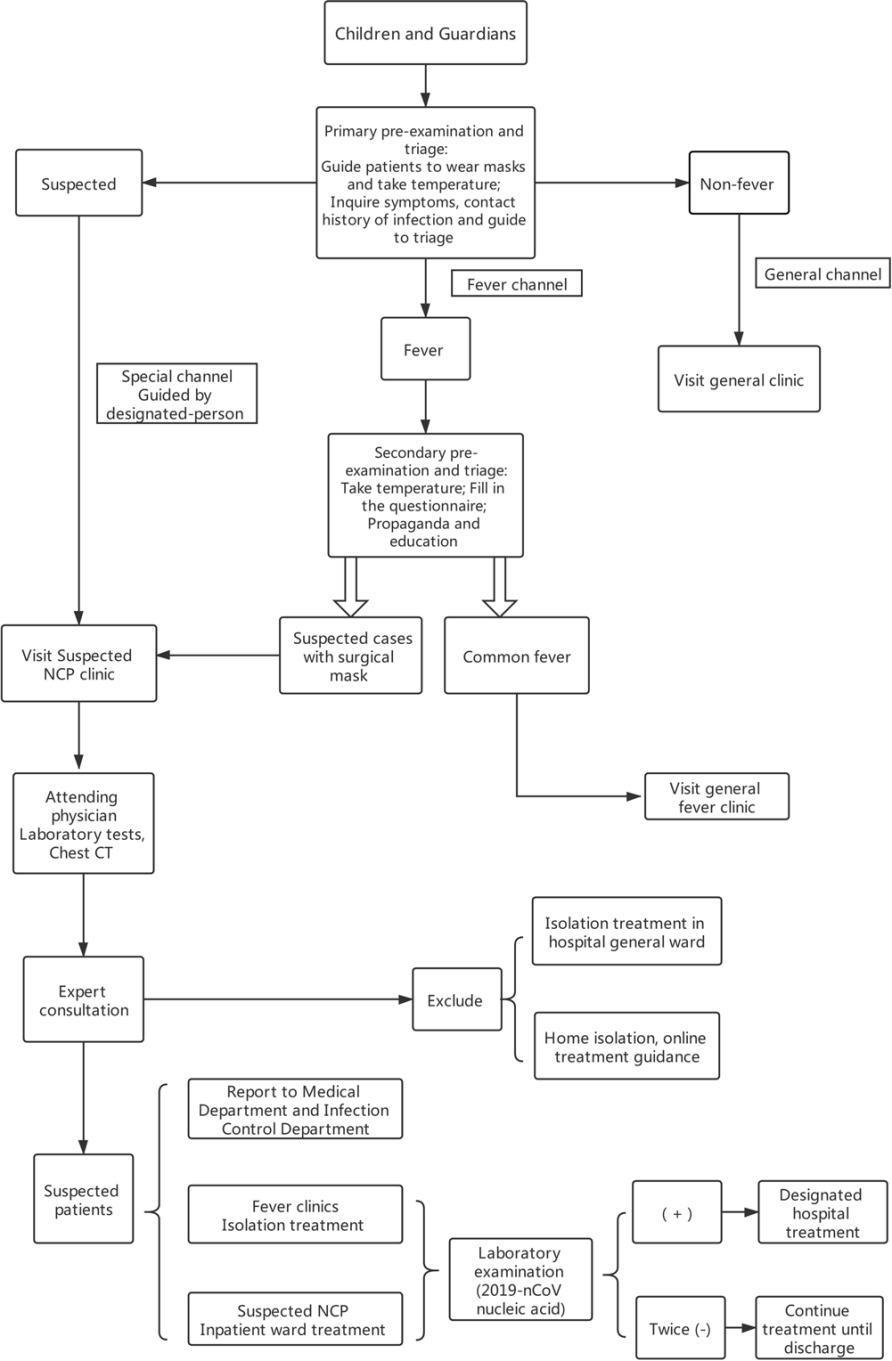
Flow diagram of febrile children diagnosis process.

1. Establish hospital-level, pre-examination and triage stations at all levels of hospitals to conduct pre-examination and subdiagnosis of children, which is called *primary pre-examination and triage* (Figure 1).
2. Four tasks should be completed^4^: ①Guide to wearing mask properly; ②Take temperature; ③Inquire symptoms and epidemiological history; ④Divide the patients into fever, non-fever, and suspected children and guide them to the fever clinic, non-fever clinic, and suspected NCP clinic, respectively, according to body temperature and epidemiological history.
3. Epidemiological history is an important evidence for early identification and diagnosis of children infected with 2019-nCoV. During pre-examination and triage, we should pay special attention to investigating the epidemiological history of parents and the situations of those who have contacted the child. School-age children should especially investigate whether there are children with a fever among their classmates. It is necessary to strengthen parents’ initiative to report suspected children through missionary education and strengthen investigations on cluster outbreak of families and residential communities (villages) and epidemiological history of family members (including children and caregivers).

*Family cluster onset*: 2 or more family members (including children and caregivers who come to the clinic) showing respiratory symptoms within 2 weeks, and 1 or more are diagnosed with 2019-nCoV infection or suspected.

*Residential community (village) cluster onset*: the community (village) where the children and the caregivers live, especially the building and apartment, and occurrence of 2 or more people showing respiratory symptoms in the past 2 weeks and diagnosed with 2019-nCoV infection.

Epidemiological history of family members (including children and caregivers) should include (1) travel or residence history in Wuhan city and surrounding areas or other areas with confirmed cases within 14 days prior to disease onset; (2) history of close contact with a 2019-nCoV-infected case (tested positive for 2019-nCoV nucleic acid) within 14 days before onset; (3) history of contact with patients with fever or respiratory symptoms who come from Wuhan and neighboring areas or communities with confirmed cases in their own province within 14 days before the onset; and (4) history of contact with people who are related with a cluster outbreak.^4,5,13,14^

1. After primary pre-examination and triage, children whose temperature is normal and patients not meeting any of the above epidemiological history are registered through the general channel (which can be set as a green sign) and go to the general clinic; febrile children enter the fever clinic through the fever channel (yellow sign can be set); and suspected children and their family members should be guided by a designated person to enter the diagnosis area of suspected cases for isolation and treatment through a special channel (red sign can be set).^15-17^
2. Staff responsible for pre-examination, triage, and guidance adopt primary protection,^18^ including performing hand hygiene and wearing medical surgical masks, disposable surgical gloves, isolation hats, work clothes, and isolation gowns. All medical personnel involved should comply strictly with the Medical Personnel Hand Hygiene Standard. An effective alcohol-based, quick-drying disinfectant can be selected; under special conditions, chlorine or hydrogen-peroxide-based hand disinfectants can also be used; when there is visible dirt, you should wash hands with sanitizer and running water, and then disinfect. When the skin is contaminated, we should remove the pollutants immediately, and then disinfect with 0.5% iodophor or hydrogen peroxide disinfectant for more than 3 minutes with a disposable absorbent material, and then wash with water; when the mucosa is contaminated, it should be rinsed with a large amount of normal saline or disinfected with 0.05% iodophor.

#### Secondary Pre-Examination and Triage

1. At the entrances of febrile children clinics and general clinics, secondary pre-examination and triage stations should be set up, respectively, and children who come to this area will be pre-examined and triaged again by a pediatrician.
2. Two pediatricians equipping with knowledge of prevention and control of NCP should be assigned to the pre-examination and triage station, and all should adopt primary protection.^13^
3. The main tasks include ① Taking the temperature again; ② Filling in the questionnaire of epidemiological history; ③ Making propaganda and education (publicize mainly the precautions to prevent cross-infection during the visit); and ④ Assessing whether the child is eligible for a suspected case and condition of 2019-nCoV infection.
4. In the secondary pre-examination and triage, if a suspected case is found, the child and his families need to be instructed to wear medical surgical masks and will be guided by a triage staff to a suspected infection area for isolation and treatment through a special channel.^13,16,17^ At the same time, according to the current epidemic situation, the suspected children are divided into 3 levels^6^:
  1. High risk: children and caregivers from families at home medical observation with suspected cases; family cluster onset with unexplained causes; or in close contact with suspected or confirmed cases within 14 days prior to onset.
  2. Intermediate risk: clustered occurrence of NCP in residential communities (villages).
  3. Low risk: residential area is not an epidemic area.
5. Also in the secondary pre-examination and triage, if the child is in critical condition with obvious respiratory symptoms, blood oxygen saturation < 92%,^11,19-21^ and epidemiological history (high risk, intermediate risk), we should treat it as suspected NCP for emergency treatment, when NCP cannot be excluded.

### Strengthen All Aspects of the Treatment Process, Early Diagnosis, Early Isolation, and Early Treatment

#### Treatment Procedures for Children With Common Fever

1. Children with common fever are numbered in the registered order in the general fever clinic. After the doctor calls the number, an accompanying person takes the child to the clinic for treatment.^16,17^ Parents who check the examination list and the results of laboratory tests should be guided and approved by the triage staff before entering the clinic.
2. The doctor responsible for the consultation must be an attending pediatrician with a rich clinical experience or a higher-level pediatrician. We must check the Children Pre-Examination Triage Registration Form filled out by the parents during the consultation and further find the clues of the suspected cases. After a detailed physical examination and consultation, the pediatrician analyzes the condition comprehensively, based on the medical history, and selects necessary laboratory examinations, such as blood routine, C-reactive protein, mycoplasma pneumoniae, chlamydia pneumoniae, erythrocyte sedimentation, nasopharyngeal swabs, and chest X-rays. Finally, a comprehensive judgment is made to further determine whether there are signs of 2019-nCoV infection. For patients whose infections can be identified as being caused by other pathogens, the possibility of 2019-nCoV infection can be initially ruled out, and appropriate treatment should be performed according to ordinary fever.
3. Medical personnel adopt primary protection.^18^ Throw disposable tongue depressors for oral examination into a covered medical waste bin (step on and open the cover, under physician’s work desk) after use. Examination tools, such as stethoscopes and percussion hammers, should be disinfected with quick-drying disinfectant before and after contact with children.^22^

#### Diagnosis Procedures for Non-Fever Children

Most of the procedure for treating non-fever respiratory and digestive tract diseases is the same as that for children with common fever. However, the physician should select the necessary laboratory examination after a comprehensive analysis of the patient’s condition and epidemiological situation. Over-examination must be prevented to avoid increase of the risk of cross-infection in children.

#### Diagnosis Procedures for the Suspected Children

1. Establish diagnosis and treatment area of the suspected NCP. The area includes consultation rooms and isolation treatment rooms. Isolation treatment rooms are provided with ultraviolet and air disinfection, emergency vehicles, and drugs according to the secondary protection.
2. The suspected child found in pre-examination and triage is guided by designated persons into the diagnosis and treatment area of suspected NCP through the special channel and enters a single-room isolation room for treatment. Younger child should be accompanied by a parent wearing a surgical mask.^4-6,13,16,17^
3. Protection of medical personnel. At least 1 attending physician, 1 resident, 1 supervisor nurse, and 1 nurse with clinical experience are expected to be involved in a suspected NCP clinic. All of them adopt secondary protection.^13,18,20,22^ They enter the sterile area through the employee-only channel and then wear medical protective masks (N95 masks or higher protection level), disposable hats, and work shoes in turn after carefully washing hands; wear work clothes before entering a potentially contaminated area (those with damaged skin or suspected injuries should wear gloves); and take off work clothes, change into protective clothing and wear disposable hats and medical protective masks, protective screens or goggles, disposable gloves, work shoes or rubber boots, and waterproof boot covers for the second time before entering the contaminated area.^22^ Stethoscopes, thermometers, sphygmomanometers, and other medical instruments and care products are cleaned and disinfected after applied.^13,22^

#### Collecting Medical History of Suspected Children

Medical history collection should be comprehensive and specific, including (1) epidemiological history of family members (including children and caregivers) – special attention should be paid to the presence of family cluster onset; and (2) clinical manifestations: fever, cough, sore throat, runny nose, diarrhea, nausea, vomiting, fatigue, general soreness, dizziness, headache, abdominal pain, and inquiring carefully the fever type and frequency of antipyretic drugs used, symptom improvement after medication.

#### Physical Examination of Suspected Children

Physical examination requires special attention: (1) heart rate, respiration: at least 1 minute for each auscultation; (2) whether the respiratory rate is consistent with the pulmonary signs; (3) whether there is hypoxia and need to inhale oxygen; if necessary, combine with the monitoring result of blood oxygen saturation to judge.

#### Laboratory Examination of Suspected Children

1. Routine examination: blood routine and C-reactive protein, if necessary, urine and fecal routines
2. Common respiratory pathogen examinations: mycoplasma pneumoniae antibody, chlamydia pneumoniae antibody, A and B nasopharyngeal swabs, respiratory syncytial virus, adenovirus
3. Special laboratory examinations: liver and renal function, myocardial enzymes, blood smear, erythrocyte sedimentation rate, coagulation function, D-dimer
4. Chest imaging: chest CT examination is required for suspected children^2,4,20,22^
5. Other tests: choose according to the condition

The above examinations were performed in the hospital’s special fever clinic. The collected specimen should be placed in a suitable freeze-proof sampling tube with a screw cap attached with a gasket inside and be tightened. The sample number, type, name, and sampling date are recorded on the container. Place the sealed specimen in an appropriately sized plastic bag. Each bag contains 1 specimen, written with “suspected NCP specimen,” and sent to the NCP examination room by a designated person.

## PRINCIPLES OF DIAGNOSIS, CONDITION JUDGMENT, AND TREATMENT OF SUSPECTED INFECTIOUS CHILDREN IN OUTPATIENT SERVICE

### Further Diagnosis of Suspected Infectious Children in Outpatient Service

The clinicians responsible for the diagnosis and treatment should make the following further diagnosis of suspected cases based on the epidemiological history, clinical manifestations, and laboratory and imaging results:

1. Suspected case: children who meet any of the criteria mentioned previously at pre-examination and triage and epidemiological history, and those who meet 2 of the following 3 clinical manifestations and laboratory test results may become suspected cases^5,6,19,21^:
  ① Fever, obvious respiratory symptoms, shortness of breath, decreased peripheral oxygen saturation, or digestive tract symptoms such as nausea, vomiting, abdominal discomfort, and diarrhea
  ② Laboratory tests: normal or decreased white blood cell count, decreased or progressively decreased lymphocyte count, normal or slightly elevated CRP
  ③ Chest-imaging findings showing signs of NCP
2. Clinical diagnosis of NCP: children who meet the following 4 criteria simultaneously may become cases diagnosed clinically as NPC: obvious clinical manifestations; cluster onset (family or participate in other parties); with radiologic manifestation of NCP; 1 person has been diagnosed with 2019-nCoV infection (tested positive for2019-nCoV nucleic acids) in the family or contacts. The diagnosis is further confirmed when the nucleic acid test or the 2019-nCov antigen test (immunofluorescence microscopy) shows positive. Among the previous criteria, if there are radiologic features, 2019-nCoV infection should be diagnosed. NCP imaging features include: in the early stage of disease, chest images show multiple small plaques and interstitial changes, which are obvious in the lung periphery, and further deteriorate to bilateral multiple ground-glass opacity and/or infiltrating shadows. Lung consolidation may occur in severe cases. Pleural effusion is uncommon.^4-6,19,21^
3. Non-compliance with suspected 2019-nCoV infection: meet the exclusion requirements of suspected cases: 2 consecutive nucleic acid tests of 2019-nCoV were negative (sampling time interval of at least 24 hours), and the new coronavirus-specific antibodies IgM and IgG were still negative 7 days after onset.

### Condition Judgment of Suspected Children

According to medical history, physical examination, and other examination and test results, the suspected children are classified into the following 4 types^5,6,19,21^:

1. Mild: mild cases are mainly children with upper respiratory tract infection. Clinical manifestations include symptoms such as fever, fatigue, myalgia, cough, sore throat, headache, runny nose and sneezing; gastrointestinal symptoms such as nausea, vomiting, abdominal pain, diarrhea; pharyngeal congestion, no positive signs in lungs at physical examination; with or without symptoms of upper respiratory tract infection, presenting asymptomatic infection.
2. Common: common cases are mainly children with early pneumonia. Clinical manifestations include fever and cough, early manifestations are dry cough followed by sputum cough; some with wheezing, but without obvious hypoxia such as shortness of breath; sputum or dry rales and/or wet rales can be heard during lung auscultation. Chest imaging showed signs of lung infection.
3. Severe: severe cases are common in children with NCP for about 1 week. Clinical manifestations include symptoms of respiratory failure and/or heart failure, dyspnea and hypoxic symptoms such as central cyanosis or oxygen saturation (< 92%).
4. Critical: critical cases, in addition to respiratory failure and heart failure, have severe symptoms such as shock. Children in critical condition may rapidly progress to acute respiratory distress syndrome (ARDS) and develop multiple organ dysfunction such as shock, encephalopathy, myocardial injury or heart failure, coagulation dysfunction, and acute kidney injury.

### Treatment Principles

According to the above classification, mild patients can be isolated at home; common, severe, and critical patients are admitted to the hospital; and critical cases should be immediately admitted to the intensive care unit.

1. Based on their medical conditions, suspected children should be isolated in single rooms or self-isolated at home following their doctors’ advice.^20,22^
2. The general treatment includes rest and supportive treatment; ensuring sufficient calorie and water intake; maintaining water electrolyte balance; monitoring vital signs and oxygen saturation; keeping respiratory tract unobstructed and inhaling oxygen when necessary; and measuring blood routine, urine routine, C-reactive protein, and other blood biochemical indexes. The patients with high fever should be actively controlled.^6^
3. Infection control: antiviral therapy: interferon-α nebulization: interferon-α 2–4μg/kg in 2 mL sterile water, nebulization 2 times per day for 7 days; and avoiding irrational use of antibiotics, especially in combination with broad-spectrum antibiotics.
4. Critical cases should be admitted to the pediatric intensive care unit as soon as possible.
5. The nucleic acid test of the 2019-nCoV should be reviewed and a case diagnosis confirmed. If the child’s specimen tested positive for 2019-nCoV nucleic acid, it’s a confirmed case and will be treated in the NCP ward of the designated hospital. The children will be treated in single isolation wards, under the care of professional medical staff, and their families observed and isolated. If the child’s first specimen tested negative, it should continue to receive isolation treatment, and be arranged to collect specimens for 2019-nCoV nucleic acid test again after 24 hours; if the results of the second tests are still negative, the children who have not improved significantly will continue to be treated until they are discharged. For the children whose conditions have improved, they could be separated at home and treated under the supervision of the community and remote doctors guidance^5,6,19,21^; if children’s symptoms disappear and home-isolation reaches 14 days (from the date of onset), they will be released.^5,6,13^

### Nursing Care Note

1. Personal protection is conducted strictly to prevent cross-infection during nursing operations, such as specimen collection, nebulization, and infusion.
2. Measures of single-room isolation are implemented for infected patients, as well as strengthening monitoring and patrolling to avoid accidental injuries, such as asphyxia, fall, and scald.
3. Preparations should be made, such as rescue medications, equipment, and personnel allocation for critical children.
4. Specimen collection and inspection must strictly comply with requirements of NCP prevention and control.
5. Feedback of specimen results should be responsible for a designated person.
6. Patients’ excreta and secretions should be disinfected.

### Management of Suspected Neonatal Cases

We treat newborns delivered by confirmed 2019-nCoV-infected mothers as suspected cases.^6,23^ Newborns delivered by mothers with a history of NCP infection within 14 days before and 28 days after delivery, and newborns contacted with other persons with history of NCP infection (including family members, health care providers, visitors), during the neonatal period, should be considered as suspected cases, regardless of the symptoms.^6,23,24^

When treating such patients, we should set up separate neonatal isolation wards, place the newborns in incubators, not use open rescue stations, and appoint someone to take care of them. Caregivers and pediatricians adopt secondary protection.^18,23,24^ When entering or leaving the isolation ward, hand hygiene and protective measures are required. If the newborn has no or only mild clinical symptoms, it will be transferred to the isolation and observation ward, and the duration of observation should be more than 14 days. Then, for a newborn in good general condition, if the mother is released from isolation, the child could also be released^23,24^; if the mother’ s 2019-nCoV nucleic acid test shows positive and the newborn shows severe clinical manifestations, the newborn would be transferred to the neonatal isolation diagnosis and treatment ward of the designated hospital for further treatment.^5,6,14,21,25-27^

### Management of the Caregivers and Visitors

Caregivers and contacts of suspected children should be guided to the adult fever clinic and be taken under medical observation and other necessary preventive measures.^5,6,14,21^ In the suspected NCP quarantine area, we should implement an unaccompanied system and forbid relatives to visit.

### Management of Confirmed Children

For confirmed children, if the hospital is a designated hospital, the children should be admitted to the children’s NCP isolation ward and treated according to relevant diagnosis and treatment standards of NCP. If not, the NCP transfer process should be followed strictly and the children transferred to the designated hospital for treatment.
